# Anterior insular cortex regulation in autism spectrum disorders

**DOI:** 10.3389/fnbeh.2015.00038

**Published:** 2015-03-06

**Authors:** Andrea Caria, Simona de Falco

**Affiliations:** ^1^Institut für Medizinische Psychologie und Verhaltensneurobiologie, Eberhard Karls Universität TübingenTübingen, Germany; ^2^Fondazione Ospedale San Camillo IRCCSVenezia, Italy; ^3^Department of Psychology and Cognitive Science, University of TrentoRovereto, Italy

**Keywords:** autism spectrum disorders, anterior insula, real-time fMRI, neurofeedback, instrumental learning

## Abstract

Autism spectrum disorders (ASDs) comprise a heterogeneous set of neurodevelopmental disorders characterized by dramatic impairments of interpersonal behavior, communication, and empathy. Recent neuroimaging studies suggested that ASD are disorders characterized by widespread abnormalities involving distributed brain network, though clear evidence of differences in large-scale brain network interactions underlying the cognitive and behavioral symptoms of ASD are still lacking. Consistent findings of anterior insula cortex hypoactivation and dysconnectivity during tasks related to emotional and social processing indicates its dysfunctional role in ASD. In parallel, increasing evidence showed that successful control of anterior insula activity can be attained using real-time fMRI paradigms. More importantly, successful regulation of this region was associated with changes in behavior and brain connectivity in both healthy individuals and psychiatric patients. Building on these results we here propose and discuss the use of real-time fMRI neurofeedback in ASD aiming at improving emotional and social behavior.

## Introduction

Autism spectrum disorders (ASDs) are neurodevelopmental disorders that dramatically impair interpersonal behavior, communication, and empathy (Pelphrey et al., 2002), with an estimated incidence of about 6:1000 (Chakrabarti and Fombonne, 2001; Levy et al., 2009). A systematic review of epidemiological surveys of autistic disorder and pervasive developmental disorders (PDD) worldwide indicated a median of ASD prevalence estimates of 62/10000 (Elsabbagh et al., 2012). On the other hand according to the US Centers for Disease Control the incidence increased to 1 in 68 children.^1^

ASD are mainly characterized by abnormal functioning in social communication and social interaction across multiple contexts, such as social-emotional reciprocity and nonverbal communicative behaviors used for social interaction, and by restricted, repetitive patterns of behavior, interests, or activities (Diagnostic and Statistical Manual of Mental Disorders DSM-5, American Psychiatric Association). Although the neurobiological and pathophysiological mechanisms remain obscure, clear evidence indicates that autism is a complex and heterogeneous disorder with multisystem and multigenic origin, where even identical genetic variations may lead to divergent phenotypes (Happe et al., 2006; Happé and Ronald, 2008; Levitt and Campbell, 2009; Geschwind, 2011; State and Levitt, 2011). Recent neuroimaging studies suggested that ASD are disorders characterized by widespread abnormalities involving distributed brain networks (Müller, 2007), but clear evidence of differences in large-scale brain network interactions underlying the cognitive and behavioral symptoms of ASD are still lacking. A large set of empirical results indicating decreased functional and structural connectivity of distributed brain networks supports the “under-connectivity theory” (Brock et al., 2002; Belmonte et al., 2004a,b; Just et al., 2004; Courchesne and Pierce, 2005; Geschwind and Levitt, 2007; Hughes, 2007; Casanova and Trippe, 2009; Müller et al., 2011). This theory along with functional connectivity findings suggested local over-connectivity but long-distance under-connectivity in ASD (Just et al., 2004; Courchesne and Pierce, 2005; Geschwind and Levitt, 2007; Rippon et al., 2007; Casanova and Trippe, 2009).

An underlying generalized disorder of synaptic connectivity resulting on alterations of distributed networks functioning has also been proposed as possible explanation of the manifested heterogeneity of ASD symptoms (Belmonte et al., 2004b; Bourgeron, 2009).

On the other hand, findings of postmortem and neuroimaging studies, although rather partial and sometimes contradicting (Sokol and Edwards-Brown, 2004), revealed pathological signs for ASD in the frontal lobes, amygdala and cerebellum (Amaral et al., 2008). Neurobiological theories on the underlying mechanisms of ASD, in particular those related to social behavior, mainly emphasized an impairment of selected brain regions, such as the amygdala (Baron-Cohen et al., 2000; Adolphs et al., 2001), superior temporal sulcus (STS; Pelphrey and Carter, 2008), and fusiform gyrus (Schultz, 2005). In addition, functional neuroanatomy investigations highlighted a relationship between the dysfunctional anterior insular cortex (AI) and the emotional and social impairment observed in ASD. Results of a recent meta-analysis of functional neuroimaging studies in ASD revealed hypoactivation of the right AI during several different tasks related to emotional and social processing (Di Martino et al., 2009).

The AI cortex critically contributes to emotional and social processing by supporting the neural representation of the own physiological state. Several studies demonstrated that the AI is involved in the explicit appraisal and awareness of emotional and bodily responses (Critchley et al., 2004). The AI represents a hub mediating interactions between large-scale brain networks related to the integration of externally-directed and self-directed emotional processes. Recent models further suggested that AI supports different levels of representation of current and predictive emotional states allowing for error-based learning of feeling states (Singer et al., 2009; Seth, 2013).

AI activity is engaged in the conscious representation of emotion in the self and in others (Lane and Schwartz, 1987; Hadjikhani et al., 2006), and it is thus critical for empathic behavior and interpersonal competence (Carr et al., 2003; Craig, 2003; Leslie et al., 2004; Blair, 2005; Amodio and Frith, 2006; Singer, 2006; Pfeifer et al., 2008).

Self-reported poor awareness of own and others’ feelings, both in autistic and typically developing individuals were associated with a reduced response in interoceptive insular cortex (Craig, 2003; Critchley et al., 2004). Silani et al. reported a relationship between a reduced response in the AI and poor awareness of own and others feelings in high-functioning autism/Asperger individuals (Silani et al., 2008). In a subsequent study, Bird et al. observed a reduced activation of the left AI in individuals with ASD as compared to control participants when exposed to empathic pain stimuli, and concluded that alexithymia mediates the empathy deficits in ASD (Bird et al., 2010).

In a functional integration perspective it has been proposed that dysfunctional AI connectivity may underlie the emotional and social impairment observed in patients with ASD (Uddin and Menon, 2009; Anderson et al., 2011a,b; Ebisch et al., 2011). AI hypoactivation in ASD would be related to a disconnection between AI and sensory and limbic structures that project to it, leading to a reduction in salience detection and subsequent mobilization of attentional resources necessary for guiding appropriate social behavior.

To date, although a large number of studies provided important neurobiological insights on dysfunctional brain mechanisms of ASD (Sears et al., 1994; Villalobos et al., 2005; Dapretto et al., 2006; Hadjikhani et al., 2007; Caria et al., 2011; Oristaglio et al., 2013) only few investigations exploited these results to develop novel treatments. Among these, preliminary EEG based neurofeedback studies showed promising results in mitigating cognitive and social emotional impairment in ASD (Kouijzer et al., 2009a, 2010; Pineda et al., 2014).

In parallel, increasing evidence showed that self-regulation of AI activity through fMRI feedback training is achievable in both healthy individuals (Caria et al., 2007; Lawrence et al., 2013) and psychiatric patients (Ruiz et al., 2013; Sitaram et al., 2014), and that volitional control of AI leads to changes in emotional behavior, such as self-evaluation of emotionally salient stimuli (Caria et al., 2010; Ruiz et al., 2013).

Building on these results we here propose the real-time fMRI neurofeedback approach for AI regulation with the aim of enhancing emotional and socio-communicative behavior in ASD.

We will first summarize results of previous pilot investigations showing the effects of EEG-neurofeedback in ASD, and then shortly review studies reporting abnormal functioning of AI in this population. Ultimately, we will present fMRI neurofeedback studies targeting AI in healthy participants and psychiatric patients, and discuss the possible use of this approach in ASD.

## EEG Neurofeedback in ASD

In the past decades ASD have emerged as a major public health and community challenge (Maglione et al., 2012) with high incidence (Levy et al., 2009; Elsabbagh et al., 2012), but at present evidence-based interventions that effectively treat the core symptoms of ASD are lacking. ASD pathophysiology remains unclear, and psychological interventions are currently the most common treatments (Maglione et al., 2012). Pharmacologic interventions are also used to temporarily reduce additional behavioral problems but do not target the core symptoms of ASD (Maglione et al., 2012). Despite some variability among approaches, the overall efficacy of both psychological and pharmacologic treatments is moderate.

A promising but under-examined neurobiologically-focused alternative for ASD treatment is represented by neurofeedback (Kouijzer et al., 2010). This approach permits to manipulate brain activity through instrumental (operant) conditioning (Sherlin et al., 2011). Instrumental learning of brain activity occurs by reinforcing, with positive or negative reward, the desired brain signals so as to establish a causal relationship between neural response and reinforcer (Schultz, 2000). A large body of literature on EEG-based neurofeedback demonstrated that it is possible to manipulate abnormal oscillatory brain activity in healthy individuals and patients by rewarding the inhibition or enhancement of specific neuroelectric activity (Heinrich et al., 2007; Arns et al., 2014; Strehl et al., 2014).

In individuals with ASD concurrent inhibition of theta power and enhancement of low beta power through neurofeedback training partially reduced autistic behavior (Jarusiewicz, 2002). In a pilot study Coben and Padolsky showed that EEG training aiming to weaken hyper- connectivity between posterior-frontal and anterior-temporal regions in ASD (Coben and Padolsky, 2007) resulted in an improvement of attention, visual perception, language and executive functions, and of some ASD core symptoms as assessed by the Autism Treatment Evaluation Checklist. A further similar investigation reported positive effects on behavioral, cognitive, and neurophysiological measures (Kouijzer et al., 2009b). Interestingly, the effects on social behavior and executive functions were maintained after one year (Kouijzer et al., 2009a). A recent neurofeedback study targeting the mirror neuron system via modulation of EEG signal demonstrated that successful mu rhythm suppression was associated with improvement of ASD symptoms, as evidenced by behavioral questionnaires administered to parents (Pineda et al., 2014).

Although promising most of these results were potentially biased by parents’ expectation as the outcomes of neurofeedback treatment were mainly based on parents’ evaluation. Thus, more objective behavioral and neurophysiological measures (e.g., quantitative EEG Scolnick, 2005) are necessary to confirm these observations.

Overall, these preliminary results suggest that EEG-based neurofeedback approach may lead to some improvement in social interactions and verbal and non-verbal communication skills in children with ASD, but more controlled clinical studies on larger samples are strongly needed.

## Regulation of AI Activity Through Real-time fMRI

A number of investigations have demonstrated that learned regulation of the BOLD signal is possible in brain areas related to different type of processing: sensorimotor, cognitive and emotional (Caria et al., 2012; Weiskopf, 2012; Sulzer et al., 2013). More importantly, real-time fMRI studies have shown that feedback training, besides allowing specific control of localized BOLD signal, may lead to changes in behavior (Caria et al., 2012; Weiskopf, 2012; Sulzer et al., 2013).

**Figure 1:**
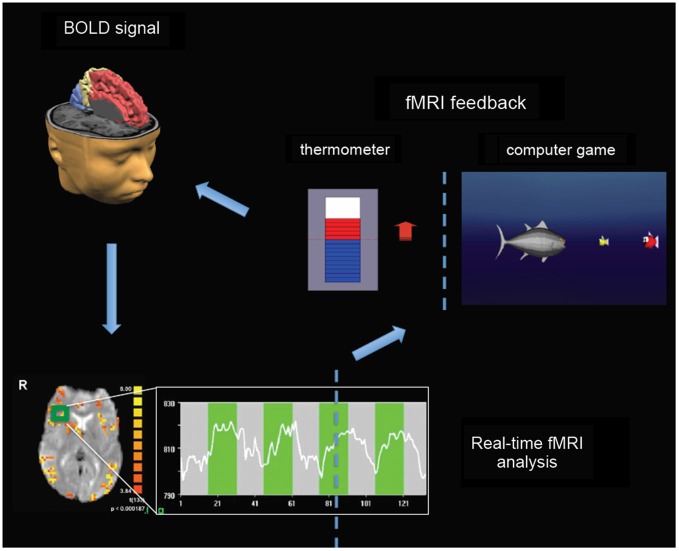
**Real-time fMRI neurofeedback setup**. BOLD signal from spatially circumscribed brain regions (e.g., the green rectangle localized in the right insula) is usually measured with fast echo planar imaging (EPI) sequences (bottom left). Real-time fMRI analysis can be performed by retrieving data online, and by performing preprocessing and statistical analysis using incremental algorithms (Caria et al., 2012). The signal time series (see graph at the bottom) of the selected regions of interest is then used to generate a visual feedback for participants (right). Information about ongoing brain activity can be depicted using thermometer bars representing the actual level of BOLD activity with respect to a baseline level, and updated typically every 1–2 s. Alternative representation of visual feedback can also be implemented using computer games or virtual reality scenarios in order to increase attention, motivation and compliance of children and adolescents (e.g., by increasing the BOLD signal a fish moves towards smaller fishes—corresponding to higher level of BOLD response—to eat them).

Most of real-time fMRI studies adopted experimental protocols consisting in a certain number of fMRI feedback sessions during which participants were trained to learn to enhance or reduce the BOLD response in selected target regions. Contingent information of BOLD signal is typically visually feedback in a continuous fashion so as to allow online monitoring and modulation of the level of activity in the target regions. The signal time series of the targeted regions of interest is then used to generate a visual feedback for the participant. Information about ongoing brain activity can be depicted using thermometer bars representing the actual level of BOLD activity with respect to a baseline level, and updated every 1–2 s (Figure 1). Alternative representation of visual feedback can also be implemented using computer games or virtual reality scenarios (Figure 1).

Participants are usually provided with some strategies (e.g., using mental imagery) that potentially permit to achieve successful regulation, though, they are also encouraged to explore different strategies and find those, explicit or implicit, most successful. Participants can be unaware of the purpose of the neurofeedback training and of the meaning of the feedback (implicit approach) (Shibata et al., 2011), in this case they would be instructed to increase the number of thermometer bars or to perform a specific visual task (Shibata et al., 2011).

In some studies the effects of self-regulation of brain activity were based on the responses to specific stimuli presented immediately after or during up-/down-regulation blocks; researchers were thus able to directly test for differences in behavior contingent to instantaneous increase and decrease of BOLD signal (Caria et al., 2010; Scharnowski et al., 2012). In other studies the effects were assessed by measuring changes in participants’ response after single or multiple feedback training runs, either in the same day or across different days (deCharms et al., 2005; Shibata et al., 2011; Scharnowski et al., 2012). In this case the observed changes in behavior were associated with a longer-term effect of learned enhancement/reduction of metabolic activity in the target regions.

Using a real-time fMRI paradigm we demonstrated that self-regulation of AI is achievable after few training sessions (Caria et al., 2007). In a consecutive study, we showed that enhanced AI activity lead to increased negative perception of aversive stimuli (Caria et al., 2010), and induced reorganization of functional brain connectivity (Lee et al., 2011).

Overall findings of real-time fMRI studies on AI indicated the suitability of real-time fMRI paradigm for clinical applications in emotional disorders. Moreover, they complemented more conventional neuroimaging studies highlighting the involvement of AI in the explicit appraisal of emotional stimuli (Craig, 2003, 2009a,b; Critchley et al., 2004).

Promising results were also shown by preliminary pre-clinical studies adopting neurofeedback protocols for AI regulation (Linden et al., 2012; Ruiz et al., 2013; Sitaram et al., 2014). In a proof-of-concept study, clinical symptoms of patients with depression significantly ameliorated after real-time fMRI training aiming to increase activity in brain regions responsive to positive stimuli, such as the ventrolateral prefrontal cortex and insula (Linden et al., 2012). A challenging study in chronic schizophrenic patients indicated their ability to learn volitional control of AI activity after real-time fMRI training (Ruiz et al., 2013). Learned control of AI affected emotion recognition so that disgust faces presented after up-regulation were more accurately detected. These results are particularly important considering that autism and schizophrenia are characterized by common etiologic and phenotypic characteristics (Barneveld et al., 2011; de Lacy and King, 2013), and that altered brain areas involved in social emotional processing were observed in both disorders (Radeloff et al., 2014).

## Self-regulation of AI in ASD

The identification of the AI as region of hypoactivation in ASD represents a key premise for proposing novel real-time fMRI experiments in autism. Previous real-time fMRI neurofeedback studies indicated that instrumental learning of AI activity affects emotional processing in healthy individuals (Caria et al., 2010; Lawrence et al., 2013) and patients with emotional disorders (Linden et al., 2012; Ruiz et al., 2013; Sitaram et al., 2014).

Considering the role of AI in emotional and social behavior, and the evidence of abnormal functionality of this region in ASD, it is reasonable to hypothesize that learned regulation of AI might potentially lead to positive clinical outcomes also in ASD. Moreover, real-time fMRI approach allowing to establish a causal link between brain activity and behavior might help to clarify the role of AI in the still unclear pathophysiology of ASD, and consequently open the way to neurobiologically-focused treatments complementing current psychosocial and pharmacological treatments.

Recent real-time functional MRI developments also indicated the possibility to provide feedback information based on dynamic functional MR connectivity (Koush et al., 2013; Zilverstand et al., 2014). Future fMRI neurofeedback investigations in ASD might directly target not simply AI but also their altered neuronal connections; for instance, participants could be trained to reinforce connectivity between AI and sensory and limbic structures.

However, several methodological issues (Sulzer et al., 2013) still need to be addressed before applying real-time fMRI neurofeedback paradigms in individuals with ASD, including how to enhance learning and motivation in emotionally sensitive patients, and how to increase behavioral effects and their translation out of the lab setting.

The role of instructions, and more in general, the strategies for achieving successful control of brain activity, might be particularly critical in ASD in light of the reported deficit in learning (Klinger and Dawson, 2001; Molesworth et al., 2005; Klinger et al., 2007; Solomon et al., 2011; Schipul et al., 2012). Impaired implicit learning was shown in individuals with low mental ages and intellectual disability during category-learning tasks (Klinger and Dawson, 2001), whereas studies involving high functioning individuals reported more intact learning abilities (Molesworth et al., 2005). Consecutive studies partially explained this difference suggesting that high functioning individuals adopt explicit strategies, for instance explicit verbal processing and reasoning, to counteract for impairment in implicit learning (Klinger et al., 2007); low functioning individuals are instead usually engaged in forced-choice tasks.

In general, unlike neurotypical individuals, showing independent mechanisms for explicit and implicit learning, individuals with ASD seem to rely on the same mental processes.

Classical fear conditioning studies in ASD also demonstrated impaired associative learning across both visual and auditory modalities (Gaigg and Bowler, 2007; Powell et al., 2012). Interestingly, it has been shown that increased explicit awareness of the learning contingencies, measured with explicit memory test, was associated with better performance in associative learning. These results also pointed to a compensatory role of explicit learning strategies, which might then be equally adopted during instrumental learning of metabolic activity.

Probabilistic reinforcement learning in ASD has also been shown to diverge from that of typically developing individuals, with ASD having further deficits in using positive feedback to exploit rewarded choices in a task requiring learning relationships between stimulus pairs (Solomon et al., 2011). Moreover, recent findings indicated that individuals with ASD have impaired ability to develop an effective reward-based working memory, and mainly rely on trial-by-trial feedback processing during learning (Solomon et al., 2015).

The evidence of impaired implicit learning and conditioned behavior implies that appropriate neurofeedback paradigm, with specific schedules of reinforcement (Rescorla, 1984; deCharms et al., 2004; Bray et al., 2007; Johnson et al., 2012), should be designed to optimize learning in ASD. Typically, real-time fMRI feedback is provided contingently to the participant’s behavior (deCharms et al., 2005; Caria et al., 2007; Shibata et al., 2011), yet, successful BOLD control in healthy individuals was shown using a delayed as well as intermittent feedback (Bray et al., 2007; Johnson et al., 2012).

On the other hand, instrumental-conditioning studies showed that learning mechanisms in ASD, although potentially altered, could be still adaptive and functional. Preliminary EEG based neurofeedback studies in children with ASD suggested that control of brain electrical activity is possible. Decreasing of excessive theta power at central and frontal regions was achieved by means of contingent feedback of brain activity, motivational reinforcement of the therapist, and no need of specific instructions.

In most of real-time fMRI studies participants were informed about potentially successful general strategies, however the role of instructions is still debated. A combination of cognitive strategies (mental imagery) and feedback information helps participants to acquire successful BOLD control (deCharms et al., 2005; Caria et al., 2010; Scharnowski et al., 2012), but whether this combination is sufficient to achieve voluntary control still remains unclear. On the other hand, studies on operant control of neuroelectric signals suggested that feedback is more important than instructions for successful cortical regulation measured with slow cortical potentials, but that instruction to imagine facilitated learning at least during the first sessions of training (Roberts et al., 1989; Birbaumer et al., 1990). It is conceivable that a combination of both explicit and implicit strategies supports learning control of metabolic activity. We think that the timely interplay of cognitive and operant strategies can facilitate brain activity control in healthy individuals and might (re)activate either impaired or dormant mechanisms in patients (Linden et al., 2012).

The use of specific instructions in individuals with ASD might be advantageous as they generally showed better performance during explicit as compared to implicit learning. However, instructions should be carefully selected in order to facilitate learning and to prevent reinforcement of dysfunctional activity and behavior. We also speculate that using appropriate pre-defined instructions might help retention of the acquired BOLD control over longer time, and possibly *response generalization* in everyday life.

Neurofeedback approach can be applied either to children or adolescents (Thompson et al., 2010; Duric et al., 2012; Steiner et al., 2014a,b); a pilot study on EEG neurofeedback feasibility in children with ASD showed that positive reinforcement and breaks including calm breathing exercises can facilitate training (Steiner et al., 2014a). However, in children with ASD, and more in general in low functioning individuals, the uncomfortable MR environment might pose serious feasibility challenges as active behaviors and vocalizations, which are frequent off-task behaviors, would detrimentally affect fMRI data. Nevertheless, specific game scenarios exploiting specific participants’ ability and interests might be implemented for both children and adolescents in order to increase attention, motivation and treatment’s compliance (Figure 1).

The effects of learned control of AI in ASD individuals can then be assessed using a variety of tests. Considering the involvement of AI in interoception (Critchley et al., 2004) and emotional awareness, which is altered in ASD (Silani et al., 2008; Bird et al., 2010), changes in interoceptive sensitivity concurrent to AI regulation might be measured.

Ratings of emotional pictures might also be used for measuring changes in emotional processing and awareness. Understanding of others’ mental states might instead be measured using tests where participants are required to associate words describing emotional states or expressions of another person’s eyes with images depicting emotional faces (Baron-Cohen et al., 1999). Pre- and post-training administration of questionnaires such as the Toronto Alexithymia Scale (TAS-20; Bagby et al., 1994) and the Davis Interpersonal Reactivity Index might also be employed for assessing outcomes related to emotional awareness and empathy, respectively.

In addition, more objective and indirect measures using physiological recordings and implicit tests (Greenwald et al., 1998; Fazio and Olson, 2003) might permit stronger conclusions on the relationship between self-regulated AI activity and emotional and social behavior.

Real-time fMRI in ASD should then entail the use of randomized, controlled investigations with specific control groups (e.g., Down syndrome and/or fully matched typical developing individuals) to assess the specificity of the potential outcomes and exclude placebo or unspecific effects. Comparisons with current available treatments for ASD, such as psychological and pharmacological interventions, should also be performed to demonstrate validity of real-time fMRI training and estimating its efficacy.

Boosting short-term, and possibly, long-term effects of neurofeedback in ASD might be even attained by combining real-time fMRI training with the administration of specific hormones or neuromodulators, such as oxytocin (Andari et al., 2010; Gordon et al., 2013; Simpson et al., 2014). It has recently been proposed that early pathophysiology in the oxytocin system by disrupting homeostatic regulation and interoception could partially account for the development of autism (Quattrocki and Friston, 2014). Moreover, recent studies demonstrated that administration of oxytocin in children and adults with ASD enhances willingness to interact socially, comprehension of affective speech, understanding of others’ mental states, and social cognition (Hollander et al., 2007; Andari et al., 2010; Guastella et al., 2010). In a real-time fMRI protocol delivery of oxytocin or neuromodulators might be, for instance, triggered by specific level of AI activity so as to benefit of concurrent biochemical and neurophysiological mechanisms.

Finally, possible issues of real-time fMRI neurofeedback approach for clinical use are the costs and accessibility of MR technology, which might limit its applicability to large cohorts of patients with ASD. To overcome this limitation more affordable alternatives to fMRI, such as optical imaging (fNIRS) and EEG might ultimately be specifically implemented and tested. For instance, assessing specific EEG correlates during real-time fMRI-based insula training might permit to build a correspondent more easily applicable EEG-based paradigm.

## Conflict of Interest Statement

The authors declare that the research was conducted in the absence of any commercial or financial relationships that could be construed as a potential conflict of interest.

## Footnotes

^1^http://www.cdc.gov/ncbddd/autism/data.html
